# Dual‐Responsive Multi‐Functional Silica Nanoparticles With Repaired Mitochondrial Functions for Efficient Alleviation of Spinal Cord Injury

**DOI:** 10.1002/EXP.70012

**Published:** 2025-02-06

**Authors:** Guibin Gao, Juanjuan Li, Yanming Ma, Min Xie, Jianxian Luo, Ke Wang, Cheng Peng, Hua Yang, Tianjun Chen, Guowei Zhang, Jiang Ouyang, Hongsheng Lin, Zhisheng Ji

**Affiliations:** ^1^ Department of Dermatology, Guangdong Provincial Key Laboratory of Allergy & Immunology The Second Affiliated Hospital of Guangzhou Medical University Guangdong Key Laboratory of Urology and Department of Urology The First Affiliated Hospital of Guangzhou Medical University Guangzhou Guangzhou Institute of Cancer Research The Affiliated Cancer Hospital and School of Biomedical Engineering Guangzhou Medical University Guangdong China; ^2^ Honghui Hospital Xi'an Jiaotong University Xi'an City China; ^3^ Department of Orthopedics The First Affiliated Hospital Jinan University Guangzhou Guangdong China

**Keywords:** diselenide‐containing mesoporous silica nanoparticles, mitochondrial functions, nanocarrier, redox‐responsive, spinal cord injury

## Abstract

Preserved/rescued mitochondrial functions have a significant effect on maintaining neurogenesis, axonal carriage, and synaptic plasticity following spinal cord injury (SCI). We fabricated an ingenious redox‐responsive strategy for commanded liberation of NADH (reduced form of nicotinamide‐adenine dinucleotide) by bioactive diselenide‐containing biodegradable mesoporous silica nanoparticles (**Se@NADH**). The nanocarrier‐embedded NADH can be liberated in a controlled pattern through the cleavage of diselenide bonds in the presence of reactive oxygen species (ROS) or glutathione (GSH). The NAD^+^ was regenerated by the reactions between released NADH and harmful ROS to antagonize mitochondrial dysfunction and increase ATP synthesis, promoting axon regeneration across SCI areas. This nanosystem increased the stability of NADH during prolonged blood circulation time, reduced the clearance rate, exhibited significant anti‐inflammatory as well as neuroprotective effects and enhanced the regeneration of electrophysiological conduction capacity across SCI areas. Importantly, **Se@NADH** suppressed glial scar formation and promoted neuronal generation as well as stretching of long axons throughout the glial scar, thereby improving actual restoration of locomotor functions in mice with SCI and exerting ascendant therapeutic effects. Targeting of mitochondrial dysfunction is a potential approach for SCI treatment and may be applied to other central nervous system diseases.

## Graphical abstract

Targeting mitochondrial dysfunction presents a potential therapeutic approach for treating spinal cord injury. We have successfully designed and synthesized a functional **Se@NADH** nanodrug system. This system offers neuroprotective and neuroregenerative benefits by modulating mitochondrial function and neutralizing reactive oxygen species, thereby reducing oxidative damage and neuronal apoptosis.

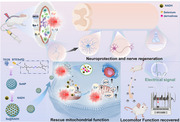

## 1 Introduction

Spinal cord injury (SCI) commonly starts from a primary injury leading to non‐reversible damage of neurons, destruction of axons and other cellular events, such as increased reactive oxygen species (ROS) levels that result in progressive secondary damage [1, 2, 3, 4]. As science and technology advance, numerous studies have made significant progress in the treatment of spinal cord injuries, which has gradually attracted widespread attention [5, 6, 7, 8]. Research into the treatment of spinal cord injuries is continuously deepening, and more therapeutic methods may be developed in the future, bringing hope to patients. Mitochondrial dysfunction is attributed to elevated excitotoxicity due to increased mitochondrial calcium buffering following SCI [9, 10, 11]. The dysfunction leads to the death of neurons and oligodendrocytes, which completes the vicious cycle of SCI [12, 13, 14]. Mitochondrial numbers and substrate utilization are critical in axon regeneration and neural stem progenitor cell differentiation [15]. Therefore, the significance of the mitochondria in SCI pathophysiology and treatment has become a topic of interest [16, 17].

Mitochondrial bioenergetics and homeostasis have a direct role in regulating cell survival following SCI, therefore, targeting the mitochondria has a direct therapeutic potential for SCI by promoting locomotor activities [18, 19]. Pharmacological interventions for mitochondrial dysfunction can suppress ROS generation and protect neurons. [20, 21] For instance, functional neuroprotection was markedly elevated by pioglitazone therapy, an antidiabetic drug, which improved mitochondrial bioenergetics after SCI. Reduced NADH (e^−^ donor) concentrations alter mitochondrial bioenergetics, such as ATP generation, by damaging the mitochondrial electron transport chain in the inner mitochondrial membrane after SCI [22, 23]. Besides, disrupted mitochondrial homeostasis such as redox disequilibrium due to increased ROS concentration or any cause may influence the ability to generate reducing substances such as NADH that aggravate secondary injury. N‐acetylcysteine amide is a modified glutathione (GSH) precursor with the ability to significantly improve mitochondrial functions by maintaining mitochondrial homeostasis, thereby playing neuroprotective roles. Physiologically, NADH is crucial in many cellular reactions such as defenses against ROS toxicity and oxidation–reduction reactions in neurodegenerative diseases [24]. In summary, NADH is essential in maintaining mitochondrial bioenergetics and homeostasis after SCI. It reverses the consequence of increased ROS production due to calcium intake in the mitochondria and has the capacity for enhanced energy production [25, 26, 27]. However, unstable NADH does not have the capacity for blood circulation to reach injured spinal cord tissues.

Selenium (Se) is an essential antioxidant that can inhibit free radical‐induced lipid peroxidation. It is a cofactor of glutathione peroxidase, a potent antioxidant enzyme that protects cells and tissues from oxidative injury. Se exerts positive clinical outcomes during the treatment of neurodegenerative diseases, implying that it has neuroprotective effects. Diselenide‐bond‐bridged silica nanoparticles can effortlessly be broken by oxygenation to form selenic acid or selenol by reducing actions in various redox circumstances [28]. As a delivery system, bioresponsive diselenide in the nanoparticles can be used to control drug release on account of the capacity of redox‐responsive biodegradability [29, 30, 31, 32]. We postulate that the diselenide‐bond‐bridged silica nanoparticles can enhance the catalysis of NADH to NAD^+^ after reacting with oxides or reductases in injured spinal cord tissues. Proliferation of electron transport chain defective cells can be enhanced by regeneration of NAD^+^ in the presence of bacterial NADH oxidase [33]. The oxidized form (NAD^+^) is an electron acceptor that can improve mitochondrial diseases [23, 34]. In addition, Se is a major component of other important antioxidant proteins such as selenoprotein S, and several other selenoproteins. Variations of selenoprotein S in injured spinal cord tissues imply that it has great potential in SCI treatment [35, 36, 37, 38].

**FIGURE 1:**
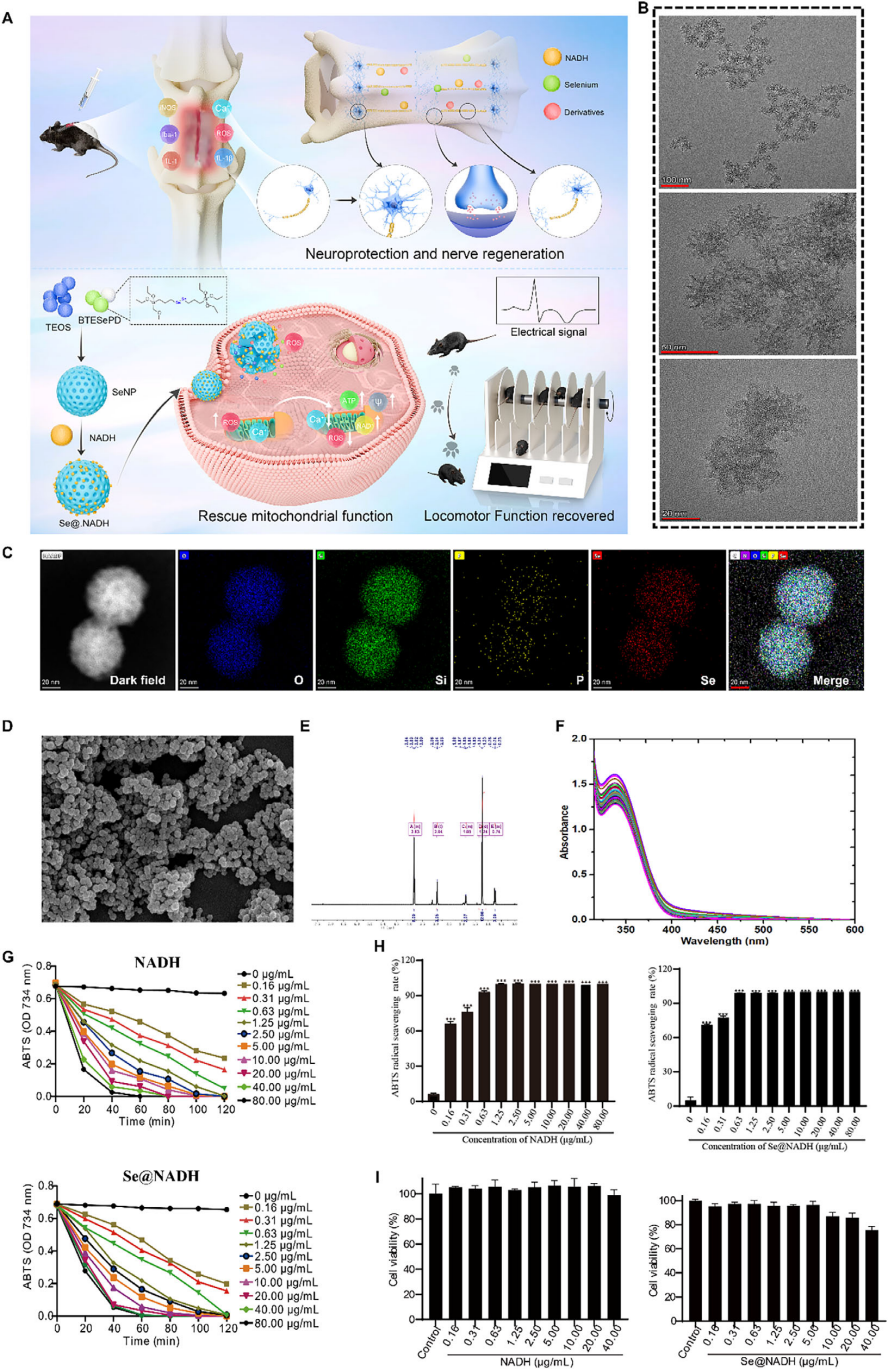
Structural characterization of Se@NADH nanomaterials. (A) Schematic illustration for the synthesis of **Se@NADH** nanotherapeutics and their neuroprotective mechanisms against SCI. (B) TEM images of **Se@NADH**. (C) Elemental mapping of **Se@NADH**. (D) Scanning electron microscopy (SEM) images of **Se@NADH**. (E) ^1^H NMR spectra of **BTESePD**. (F) UV–vis spectra of **Se@NADH** nanomaterials. (G) In vitro antioxidant activities of NADH and **Se@NADH** nanomaterials(0–80 µg mL^−1^)as determined by ABTS free radical scavenging assays, respectively (from top to bottom). (H) Quantitative analysis of ABTS results for NADH and **Se@NADH** nanomaterials in G chart. *p < 0.05, **p < 0.01, and ***p < 0.001. (I) Cell viabilities of HT‐22 cells (human neuroblastoma cells) co‐treated with different concentrations of NADH, Se@NP and **Se@NADH** nanomaterials for 48 h. *p < 0.05 and **p < 0.01.

Given the importance of Se and NADH in SCI treatment, we designed a novel redox‐triggered degradable diselenide‐bond‐bridged silica‐drug nano‐carriers for NADH co‐delivery. The diselenide‐bridged and NADH‐insertion silica nanophase materials (**Se@NADH**) with unparalleled biodegradability can be used for SCI therapy (Figure 1A). Mesoporous silica envelopment can also extend the retention time of NADH in blood and decrease the clearance rate. The NADH was discharged from nanoparticles by the destruction of diselenide bonds in the presence of ROS while NAD^+^ was regenerated by the reaction between released NADH and harmful ROS to antagonize mitochondrial dysfunction and increase ATP synthesis. These effects promoted axonal regeneration in SCI. In this study, **Se@NADH** exhibited excellent ROS scavenging activities in vitro and protected the hippocampal neurons from Sprague–Dawley (SD) rats by antagonizing mitochondrial dysfunction. The neuroprotective effects of **Se@NADH** in SCI were also explored. The nanosystem restored the motor abilities of mice with SCI by promoting voluntary signaling and neurotransmitter actions in spinal cord tissues. These findings show that **Se@NADH** enhances neural regeneration and remyelination and is a potential therapeutic option for SCI. We provide effective diselenide‐bridged and NADH‐embedded biodegradable silica nanoparticles and elucidate on neuroprotection and neuroregeneration mechanisms of the nanoparticles in SCI.

## 2 Results and Discussion

### 2.1 Synthesis and Characterization of Se@NADH

To achieve synchronous biodegradability and redox reactivity release mode in one delivery platform, a neoteric form of silica‐drug **Se@NADH** nanophase materials was designed. The diselenide group was introduced into organosilica moieties to form the mesoporous silica framework. Morphological characteristics of **Se@NADH** nanoparticles were detected by transmission electron microscopy (TEM) (Figure 1B) and scanning electron microscopy (SEM) (Figure 1D). The **Se@NADH** exhibited uniform spherical nanoparticles with diameters of ≈50 nm. Inductively coupled plasma optical emission spectrometry showed that Se and silicon coexisted while selenium density in **Se@NADH** was 5.3%. Elemental mapping of Si and N revealed that NADH was decentralized within the interior of Se@NP (Figure 1C). The resulting product exhibited desirable redox reactivity release modes. The diselenide‐bond‐bridged **Se@NADH** nanoparticles were prepared by bis[3‐(triethoxysilyl)propyl]diselenide (BTESePD) reactions with TEOS (3:2), synthesized and characterized by ^1^H NMR spectrum. Peaks were observed at 3.83 ppm, which was attributed to the methylene protons linked to oxygen atoms. The corresponding quantity of peaks at 1.22 ppm was 18, corresponding to the quantity of methyl protons (Figure 1E). Furthermore, the **Se@NADH** nanoparticles exhibited some degree of stabilities under hydrolysis for over 8 h, which increased the stability of NADH in PBS (Figure 1F).

The increased abundance of ROS during SCI results in secondary injury to the axons as well as neural systems. As an efficient superoxide scavenger, NADH has the capacity to prevent ROS‐induced neuronal damage. We assessed the ROS scavenging abilities of NADH and **Se@NADH** via ABTS, a free radical scavenging assay. In Figure 1G,H, **Se@NADH** and NADH dose‐ and time‐dependently suppressed the generation of ABTS free radicals, that was consistent with the results of DPPH (Figure S1). Meanwhile, **Se@NADH** exhibited prolonged ROS clearance ability, suggesting that encapsulation of Se@NP achieved slow NADH release. To assess the cytotoxic effects of **Se@NADH** and NADH, HT‐22 cells (Hippocampal neuronal cell line) were respectively pretreated with **Se@NADH** and NADH. Figure 1I shows that neither **Se@NADH** nor NADH exerted cytotoxic effects on HT‐22 cells, apart from the slight cytotoxic effects of **Se@NADH** at a dose of 40 µg mL^−1^. Due to its excellent free radical scavenging capacity and limited cytotoxicity, the concentration of 0.63 µg mL^−1^ was selected for the subsequent assays.

### 2.2 Dual‐Responsive Degradation of Diselenide‐Bridged Se@NADH

**FIGURE 2:**
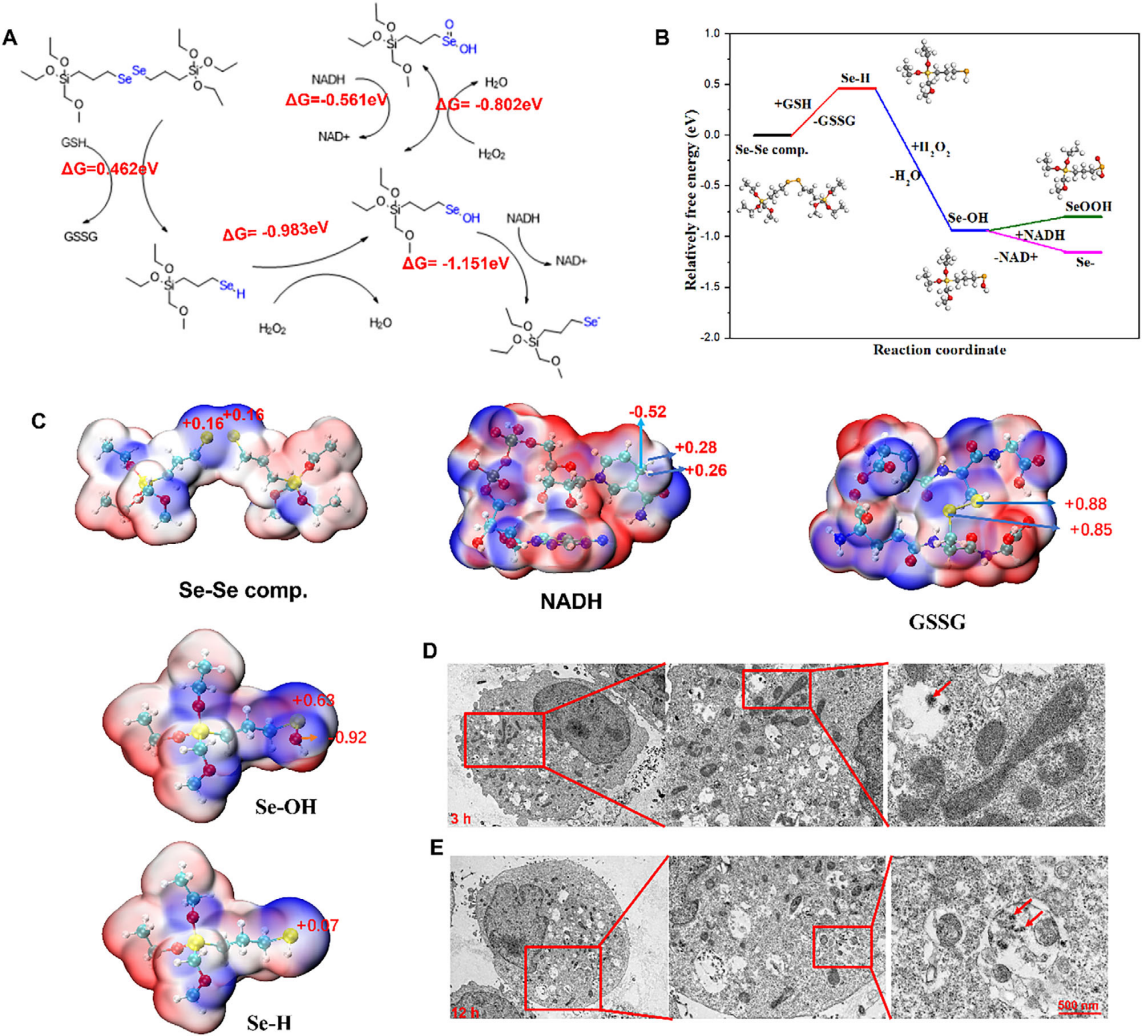
Dual‐responsive degradation and NADH release behaviors of diselenide‐bridged Se@NADH. (A) Proposed mechanisms for dual‐responsive degradation of **Se@NADH** and production of NAD^•^ radicals. (B) Calculated free energy profiles of selenium metabolites in water during reactions between **Se@NADH** and GSH or ROS. (C) Electron cloud distribution of Se–Se complex NADH and possible metabolites of selenium. (D,E) TEM image of **Se@NADH** internalized in HT‐22 cells. HT‐22 cells were incubated with **Se@NADH** (0.63 µg mL^−1^) for 3 h (D) or 12 h (E). The red arrows refer to nanomaterials **Se@NADH** in HT‐22 cell.

In the presence of an oxidizing agent or reductant, diselenide bonds can be readily disintegrated to form seleninic acid or selenol, respectively [39, 40]. Diselenide‐bond‐bridged **Se@NADH** nanoparticles have susceptible diselenide bonds that can be easily ruptured in the presence of oxidants or reductants, resulting in nanoparticle decomposition. This decomposition reaction can enable the complete release of NADH. The dual‐responsive degradation mechanism of diselenide‐bridged **Se@NADH** was hypothesized by calculating the free energy profiles of selenium metabolites [41, 42]. In Figure 2A,B, the free energy for each step was less than zero besides the degradation behavior of **Se@NADH** in GSH (Δ*G* = 0.463 ev) indicating that was a very easy crossed barrier. Ultimately, selenol was generated by diselenide reactions with H_2_O_2_, further reacting with NADH to produce NAD^+^. Electron cloud distribution of the Se–Se complex, NADH and possible metabolites of selenium prove the possibility of the reaction (Figure 2C). The degradation behaviors of **Se@NADH** nanoparticles in the presence of ROS or GSH were confirmed by TEM. The TEM images showed that after 2d of incubation in the presence of either the oxidant or reductant, the selenium‐based **Se@NADH** nanoparticles degraded into fragments of varying sizes (Figure S2). Furthermore, as shown in Figure 2D,E, **Se@NADH** were found in the cells after 3 h, and after 12 h of incubation in HT‐22 cells, the selenium‐based nanoparticles **Se@NADH** degraded into fragments. These findings imply that the diselenide bonds of the nanoparticles were cleaved by ROS and GSH to facilitate the elimination of metabolites of the nanoparticles in vivo. The introduced NADH was readily released, which accelerated the production of NAD^•^ radicals to protect the neurons and promote functional rehabilitation of the mitochondria after SCI.

### 2.3 Se@NADH Repaired Mitochondrial Functions and Downregulated ROS Levels after SCI In Vitro

**FIGURE 3:**
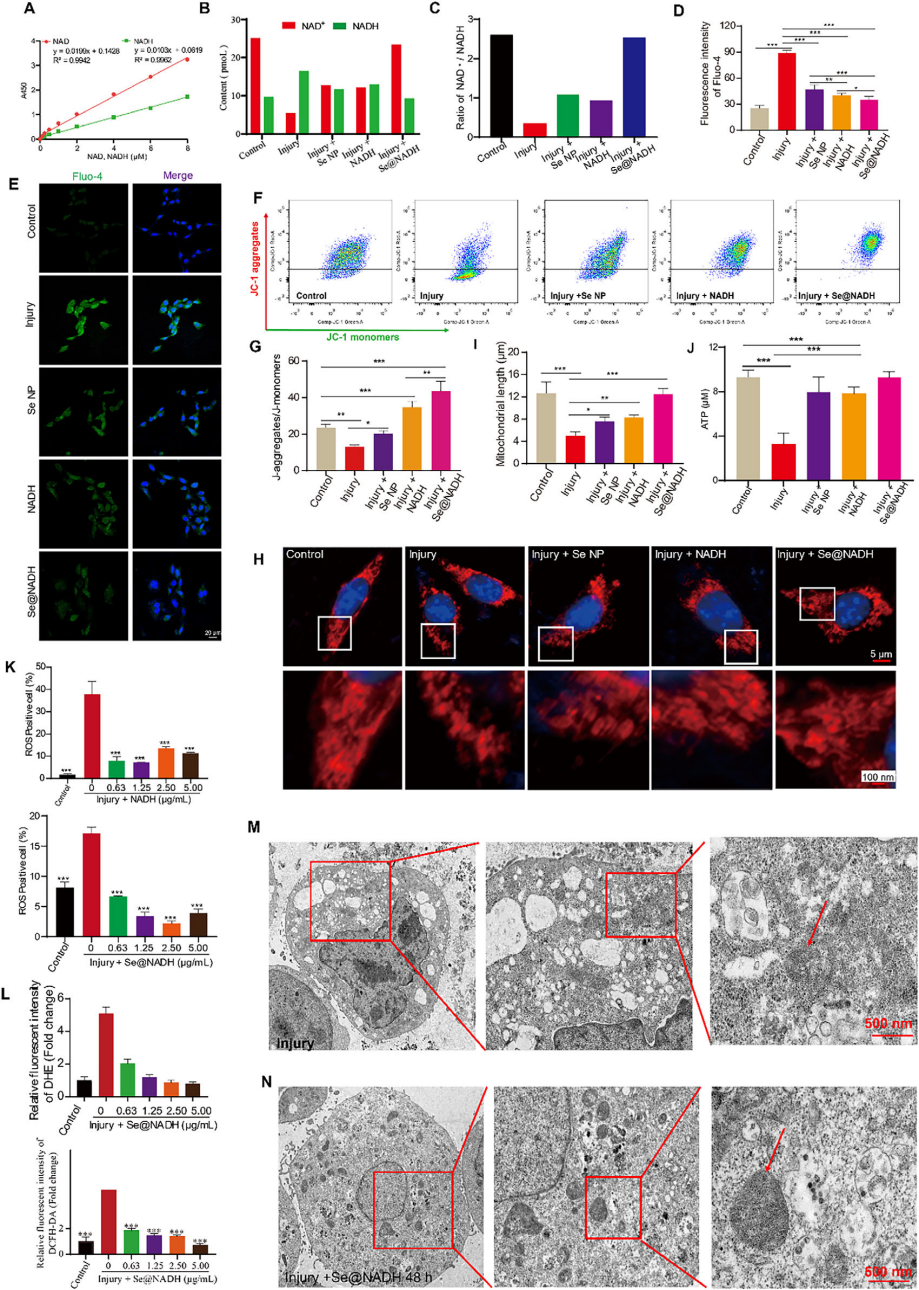
Se@NADH repaired mitochondrial functions and downregulated ROS levels after SCI in vitro. (A) Standard curve for determining the content of NAD^+^ and intracellular NAD^+^/NADH ratios. (B,C) Levels of NAD^+^ and intracellular NAD^+^/NADH ratios were determined using the NAD^+^/NADH assay kit with WST‐8. (D,E) Levels of Ca^2+^ in indicated groups were determined by the calcium ion detection test. (F,G) Mitochondrial potential in indicated groups was determined and quantified by assessing JC‐1 aggregates/JC‐1 monomers by flow cytometry. (H,I) Representative images of mitochondrial morphology, quantification of mitochondrial length and percentage of cells with fragmented mitochondria in indicated groups (Scale bar = 5 µm). (J) ATP was used to assess mitochondrial functions in HT‐22 cells. ns, *p* > 0.05; **p < 0.01; ***p < 0.001; ****p < 0.0001. Means ± SEM. (K) Quantification of ROS levels treatment with **Se@NADH** (left) and NADH (right) by DCFH‐DA in HT‐22 cells by flow cytometry in indicated groups. (L) Detection and quantification of ROS levels treatment with **Se@NADH** by DHE (left) and by DCFH‐DA (right) in HT‐22 cells by flow cytometry in indicated groups. (M,N) Representative TEM images of the mitochondria in the HT‐22 cells injured by glutamic acid (GA, 120 µM) (M) Or for **Se@NADH** (0.63 µg mL^−1^) treatment 48 h (N). The red arrows refer to mitochondria in HT‐22 cell.

The mitochondria play a vital role in SCI‐associated secondary damage via their involvement in Ca^2+^ homeostasis and ROS production [43, 44]. Mitochondrial dysfunction after SCI resulting from Ca^2+^ overload accelerates the formation of mitochondrial penetrability transition pores to result in ATP depletion and post‐traumatic apoptosis [45, 46, 47, 48]. Regeneration of NAD^+^ lengthened the lifetime of mice with mitochondrial diseases and rescued ATP levels [23, 49]. Given the significant effects of NAD^+^ regeneration in mitochondrial diseases, NAD^+^ levels and the NAD^+^/NADH ratio were characterized using the NAD^+^/NADH assay kit with WST‐8. In Figure 3A–C, **Se@NADH** significantly promoted NAD^+^ levels and NAD^+^/NADH ratio to reverse injury outcomes in HT‐22 cells. The NAD^+^/NADH ratio was increased fivefold after **Se@NADH** therapy in the injured group, consistent with results from the control group, which shows the promising capacity of the nanoparticles for NAD^+^ regeneration. Treatment with **Se@NADH** suppressed mitochondrial calcium ion (Ca^2+^) concentrations, which shows the capacity of nanoparticles to alleviate mitochondrial dysfunction (Figure 3D,E). The JC‐1 staining assay revealed significantly suppressed mitochondrial potential in injured HT‐22 cells, which was improved by **Se@NADH** treatment (Figure 3F,G). In Figure 3H,I, **Se@NADH** treatment reversed injury‐associated mitochondrial morphological changes and increased the mitochondrial length by repairing the fragmented and swollen mitochondria in HT‐22 cells. Moreover, **Se@NADH** treatment significantly increased ATP production (approximately three times; Figure 3J and Figure S3), suggesting improved respiration capacities of HT‐22 cells. Increased ROS production is associated with mitochondrial damage and plays a significant role in accelerating functional dysregulation following SCI [38, 50]. Therefore, we detected ROS levels in HT‐22 cells after 24 h of incubation in the presence of NADH and **Se@NADH**. In Figure 3K and Figure S4 and S5, NADH and **Se@NADH** treatment markedly decreased ROS levels in HT‐22 cells, which contrasts with the consequence of the injured group, as reflected by the remarkable decrease in green and red fluorescence (Figure 3L and Figure S6–S7). **Se@NADH** treatment dose‐dependently lessened the fluorescence intensity in HT‐22 cells, implying improved ROS scavenging capacities in cells. Mitochondrial damage was repaired that further demonstrated by the increased mitochondrial crista after **Se@NADH** treatment at 48 h in HT‐22 cells (Figure 3M,N). These results show that **Se@NADH** treatment alleviated ROS generation and improved mitochondrial dysfunction following SCI.

### 2.4 Protective Effects of Se@NADH Against Oxidative Stress‐Induced Damage

**FIGURE 4:**
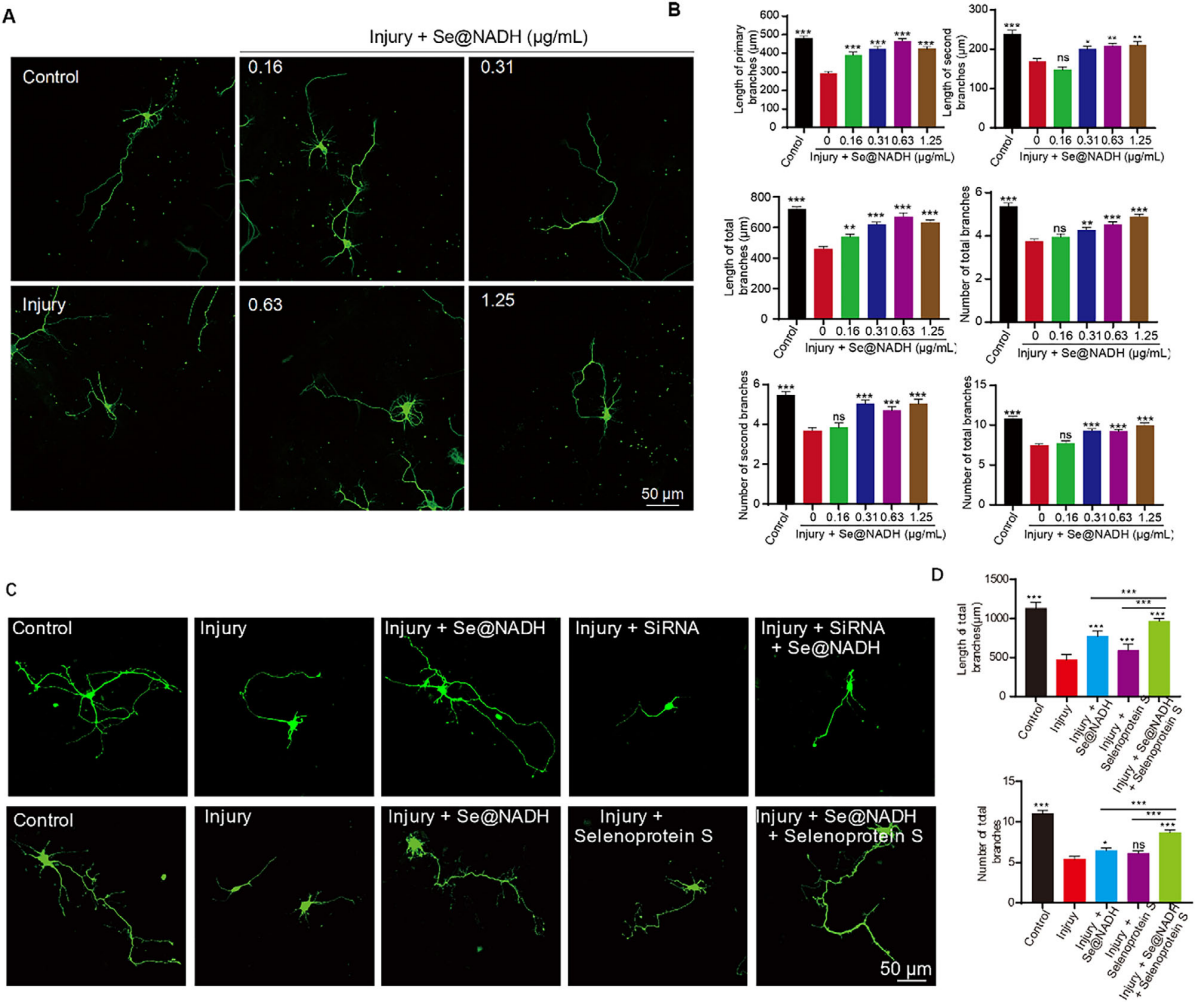
Se@NADH promoted neurite outgrowths. (A) Representative images of hippocampal neurons after co‐cultures with **Se@NADH** for 24 h. Scale bar: 50 µm. (B) Above: Total lengths of primary and secondary branches, revealing that **Se@NADH** promoted neurite outgrowths (*n* = 15/group). Below: Total number of primary and secondary branches, revealing that **Se@NADH** promoted branch formation (*n* = 15/group). Data are expressed as means ± SD for *n* = 3. *p < 0.05, **p < 0.01, and ***p < 0.001. (C) Above: Representative images after co‐cultured with **Se@NADH** for 24 h in knockdown selenoprotein S of hippocampal neurons; Below: Representative images after co‐cultured with **Se@NADH** for 24 h in overexpression selenoprotein S of Hippocampal neuron. Scale bar: 50 µm. (D) Above: The total length of primary and secondary branches, revealing that **Se@NADH** promotes neurite outgrowth in overexpression selenoprotein S of Hippocampal neuron (*n* = 15/group). Below: The total number of the primary and secondary branches, revealing that **Se@NADH** promotes branch formation in overexpression selenoprotein S of Hippocampal neuron (*n* = 15/group). Data are means ± SD, based on triplicate experiments. *p < 0.05, **p < 0.01, and ***p < 0.001.

To intuitively understand the protective effects of **Se@NADH** after SCI in vitro, neuronal morphologies as well as the total number and lengths of branching neurites on hippocampal neurons were investigated. Figure 4A shows that healthy neurons exhibited a filamentous network while those in the injury group exhibited severely destroyed fragments. Damaged neurons were repaired using specific concentrations of **Se@NADH**. The total number and lengths of branching neurites on hippocampal neurons increased with increasing **Se@NADH** concentrations, suggesting that **Se@NADH** protected the hippocampal neurons from oxidative stress‐induced injury (Figure 4B). Particularly, 0.63 µg mL^−1^ **Se@NADH** exhibited an excellent ability to promote nerve growth. Comparable findings were also obtained after treatment with NADH (Figure S8). The morphological improvements further indicated that **Se@NADH** protected hippocampal neurons and accelerated neuronal growth following SCI by repairing mitochondrial functions and downregulating ROS levels. The significance of selenoprotein S in SCI has been reported. Boosting selenoprotein S levels alleviates SCI‐associated secondary injury by reducing inflammation and oxidative damage [36, 38]. Given the essential role of selenoprotein S in SCI‐associated secondary injury, the morphology of neurons and total number as well as length of branching neurites on hippocampal neurons following knockdown and overexpression of selenoprotein S were investigated (Figures S9 and S10). Selenoprotein S knockdown in hippocampal neurons significantly decreased the total number and length of branching neurites while neural morphology was not improved (Figure 4C). Conversely, during the overexpression of selenoprotein S in hippocampal neurons, the morphology was further improved and the total number as well as length of branching neurites on hippocampal neurons increased, revealing the essential role of selenoprotein S in SCI‐associated secondary injury (Figure 4D). In summary, selenoprotein S was regulated by **Se@NADH** to protect hippocampal neurons from oxidative stress‐induced injury.

### 2.5 Se@NADH Promoted Motor Function Recovery

**FIGURE 5:**
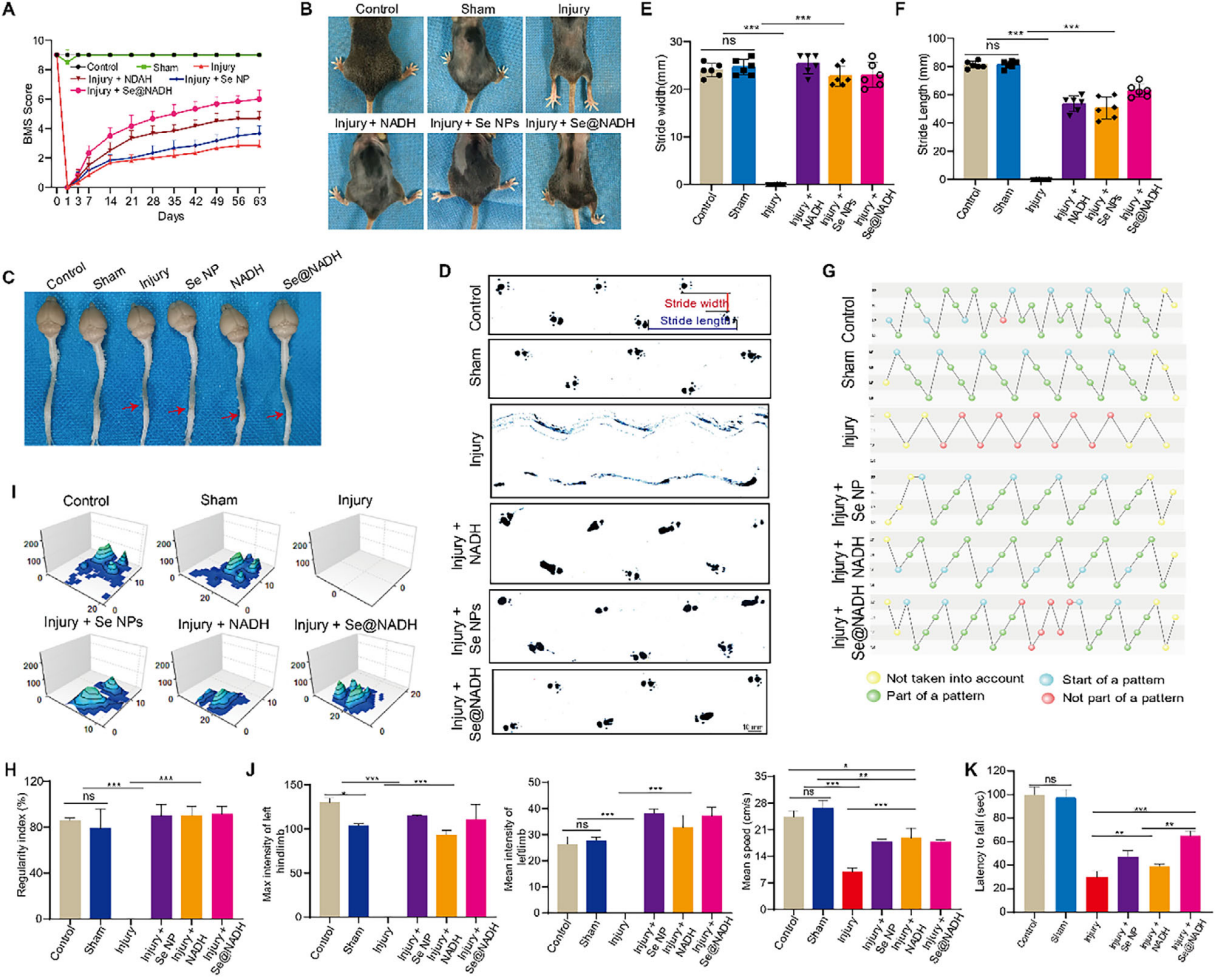
Se@NADH promoted motor function recovery. (A) BMS was used to functionally grade mice in different groups up to 9 weeks post‐injury. (B) Typical images of feet in SCI rat models in control, sham, injury, injury + SeNP, injury + NADH, or injury + **Se@NADH** groups. (C) Images of spinal cords of rats in the control, sham, injury, injury + SeNP, injury + NADH, or injury + **Se@NADH** groups at 9 weeks post‐injury. Red arrows indicate lesion sites. (D–F) Representative footprints and stride length analysis of mice at 9 weeks post‐injury. (G,H) CatWalk system for normal step‐pattern analysis of mice at 9 weeks post‐injury. (I,J) Three‐dimensional graphs of LH and RH prints boxed and footprint intensity analysis of mice at day 9 weeks post‐injury. RH, right hindlimb; LH, left hindlimb. (K) The roller test was used for functional measurement of mice at 9 weeks post‐injury.

To investigate the potential effects of **Se@NADH** in SCI, mice models of contusion SCI were established. After the treatment of animal models with **Se@NADH**, a series of animal behaviors were examined at specified times. In Figure 5A, the BMS behavioral measurement revealed that **Se@NADH** injection resulted in higher scores of hindlimb movement after SCI that effectively reversed the effects of the injury. This result was further confirmed by the increase in mice weights (Figure S11A). Further, the **Se@NADH** treated mice exhibited good therapeutic outcomes, compared to those treated with NADH. In addition, continual weight‐sustained plantar walks and imbalanced posterior limbs with forelimbs were found following treatment with **Se@NADH**. The SCI mice models had posterior limbs that were incapable of weight support (Figure 5B). Moreover, the lesion sizes of the spinal cord in mice following the **Se@NADH** therapy were smaller than in treatment with saline (Figure 5C). Stride lengths of mice in the **Se@NADH** treated group were significantly longer than those of SCI mice, however, apart from the injury group, differences in step widths in all treatment groups were comparable (Figure 5D–F). The CatWalk system was used for a detailed quantitative assessment of motor functions involved in the coordination and balance of SCI mice models (Figure 5G,H) [51, 52]. The 3D footprint intensity tab portrays the print intensity of hindlimbs of SCI mice models to comprehensively assess walking motion (Figure 5I,J). Differences in intensity and speed of hindlimbs between **Se@NADH** therapy and injury groups were significant. The entire loss of hindlimb movement occurred in the injury group, on the contrary, there was a relatively rapid renovation of locomotion functions in the **Se@NADH** treated group. Findings from roller and open field tests were comparable to those of the CatWalk system (Figure 5K and Figure S11B). In summary, **Se@NADH** relieved secondary damage and facilitated the renovation of the locomotor abilities of SCI mice models.

### 2.6 Axonal Remyelination, Synapse Formation in Lesion Areas, and Electrophysiological Improvement by Se@NADH Following SCI

**FIGURE 6:**
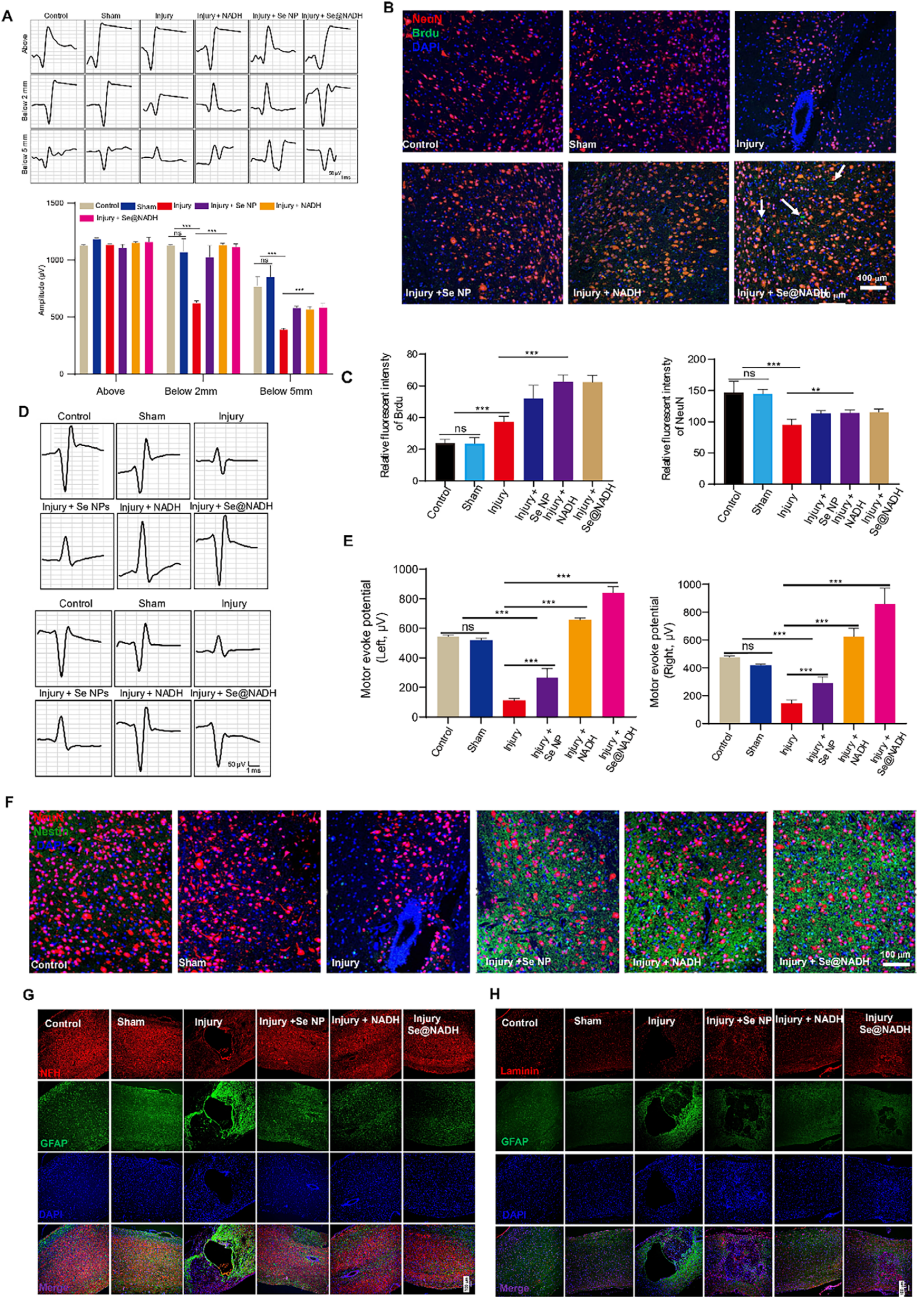
Axonal remyelination, synapse formation in lesion areas, and electrophysiological improvement by Se@NADH following SCI. (A) Representative individual electrophysiological traces at 2 and 5 mm below lesions relative to the potential above the lesion in different treatment groups or equivalent distance in control groups. **p < 0.005, ***p < 0.0001. (B,C) Immunofluorescence images of spinal cord tissues stained with anti‐BrdU (red) and anti‐NeuN antibodies (green) to label the regenerating neurons. Scale bar: 100 µm. (D,E) Representative MEPs and amplitude analysis of mice in different groups up to 9 weeks post‐injury. (F) Immunofluorescence images of spinal cord tissues stained with anti‐nestin antibody (green) and anti‐NeuN antibody (red) to label eNSPC recruitment. Scale bar: 100 µm. (G) Immunofluorescence images of spinal cord tissue stained with NFH–GFAP co‐staining of the spinal cord tissues from different treatment groups and their quantitative analysis. Scale bar: 100 µm. (H) Immunofluorescence images of spinal cord tissue stained with Laminin‐GFAP co‐staining of the spinal cord tissues from different treatment groups. Scale bar: 100 µm.

We conducted electrophysiological tests at 9 weeks after SCI to broadly assess the effects of **Se@NADH** treatment on the functional recovery of SCI mice models. A stimulating electrode was made above the lesion two spinal sections, as well as document were put below the lesion two spinal sections (Figure 6A). Postsynaptic cord dorsum potentials were largely alleviated by acute SCI, resulting in minimal potentials below the lesion. In **Se@NADH**‐treated animals, stimulation above the lesion evoked a higher response that was much stronger than that of the injury group. The Se@NADH treatment group exhibited more robust response amplitudes compared to both the sham and control groups. However, amplitudes measurably recovered after **Se@NADH** treatment, relative to the injury group. These findings indicate that **Se@NADH** resulted in a functional recovery by reestablishing spoiled neural circuits and propagation of descending impulses across SCI.

Neural regeneration was also assessed by staining anti‐BrdU and anti‐NeuN antibodies, which represent newborn cells and mature neurons, respectively. In Figure 6B,C, the NeuN fluorescence was limited, which is attributed to the reduction of neuron numbers in the injury group. Meanwhile, the NeuN staining of some neurons seemed to be not inside the nucleus in the sham and injury groups. Therefore, expressions of NeuN were affected by pathological changes in the damaged area of spinal cord. However, the number of neurons in the **Se@NADH**‐treated group significantly increased, suggesting that the neuroprotective intervention enhanced the survival of neurons during secondary damage. Positively stained BrdU and NeuN represented regenerated neurons in the **Se@NADH**‐treated group. The motor‐evoked potentials (MEP) produced during inflection and spreading motions of the ankle joint in mice indicated the capacity of **Se@NADH** to activate sensorimotor propagation pathways. Amplitudes of MEPs in the injury groups were evidently inferior to that of the **Se@NADH**‐treated group, as revealed by stimulation of the motor cortex, regardless of the left or right brain (Figure 6D,E). Recruited endogenous neural stem/progenitor cells (eNSPCs) to a lesion site improves neural regeneration. Therefore, the anti‐nestin antibody was used to assess eNSPC recruitment. In Figure 6F, the number of nestin‐positive and NeuN‐positive cells in the **Se@NADH**‐treated group was higher than those in saline‐ and NADH‐treated groups. These results show the capacity of **Se@NADH** for eNSPCs recruitment.

Double‐staining of anti‐NFH and anti‐MBP antibodies was also used to assess the quantities of myelinated axons and remyelination of regenerated axons. In Figure S12, **Se@NADH** treatment improved axonal myelination in the injury site, relative to NADH or sham groups, which shows the potential of **Se@NADH** to facilitate remyelination. These findings were confirmed by hematoxylin and eosin (H&E) staining and Luxol fast blue (LFB)‐staining (Figure S13). Glial scars can suppress the long‐distance extension of axons into damaged tissues and inhibit motor function rehabilitation [53]. The shapes of glial scars as well as the survival and regeneration of axons were evaluated by double‐staining with anti‐GFAP and anti‐NFH antibodies. In Figure 6G, treatment with Se@NADH reversed the decrease in NFH intensity and enhanced GFAP expressions around the injury site, indicating that the therapy facilitates nerve fiber rebirth and decreases the shapes of reactionary astrogliosis as well as a glial scar in vivo. Moreover, axon regeneration decreased glial scar formation after Se@NADH treatment, as revealed by double‐staining of anti‐GFAP and anti‐laminin antibodies, which are known axon‐supportive substrate molecules in SCI (Figure 6H).

### 2.7 Anti‐Inflammatory Effects of Se@NADH and Their Ability to Reduce Apoptosis In Vivo

**FIGURE 7:**
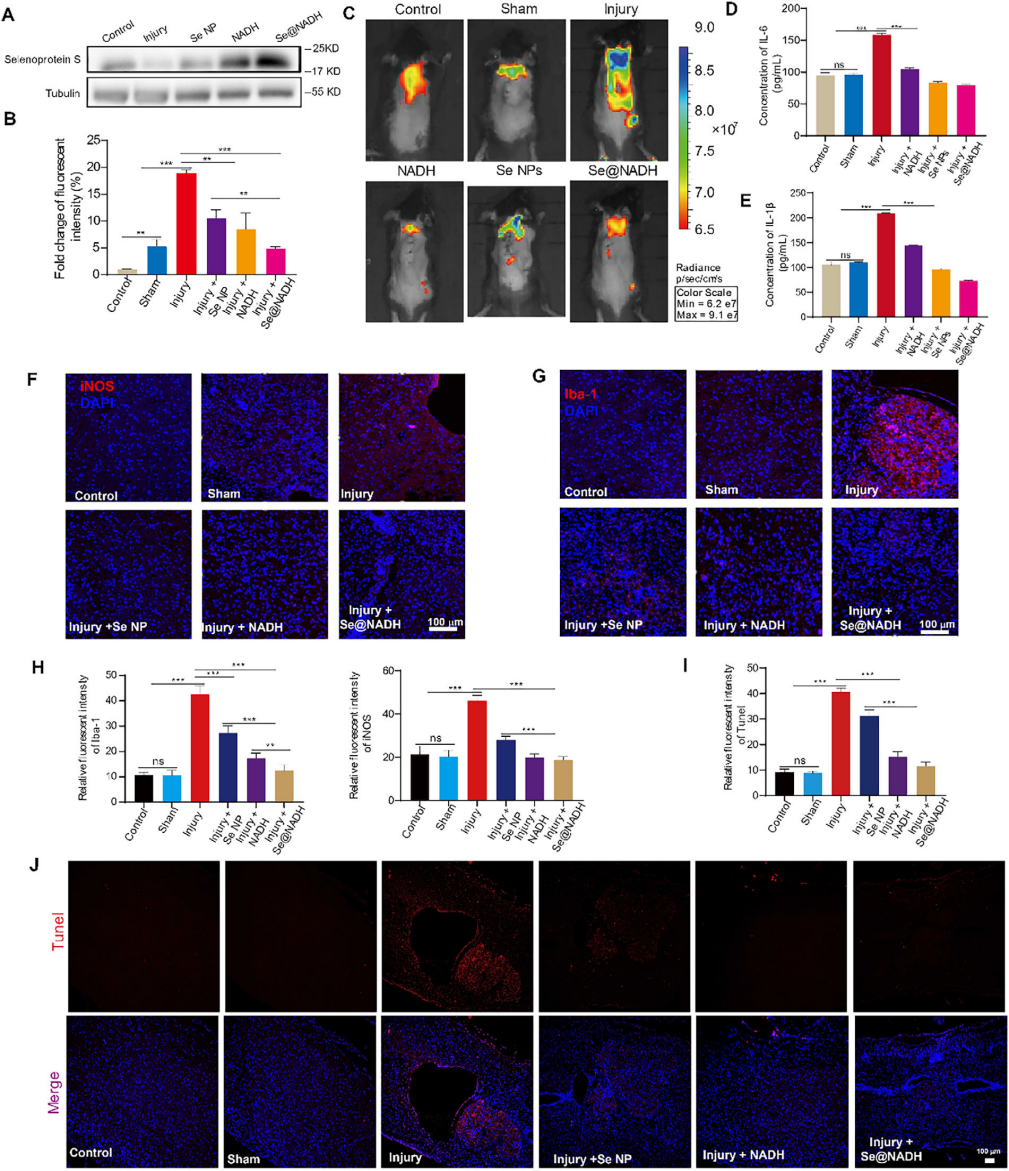
Anti‐inflammatory effects of Se@NADH and its ability to reduce apoptosis in vivo. (A) The protein level of selenoprotein S in spinal cord tissue after treatment with different treatments. (B,C) Representative images of L‐012‐stained oxidative stress in mice after different treatments (B), quantitative analysis of the intensity of oxidative stress in mice (*n* = 4, (C)). (D,E) Quantitative analysis of IL‐6 and IL‐1β protein levels by Elisa kits in spinal cord tissues of mice subjected to different treatments. (F,G) Immunofluorescence images of spinal cord tissues when spinal cord tissues were stained with anti‐INOS (F) and anti‐Iba‐1 (G) antibodies, respectively. Scale bar: 100 µm. (H) Quantitative analysis of the fluorescence intensity of anti‐INOS and anti‐Iba‐1 in spinal cord tissues of mice receiving different treatments. (I,J) Immunofluorescence images and quantitative analysis of spinal cord tissue stained with TUNEL–DAPI co‐staining of the spinal cord tissues from different treatment groups. Scale bar: 100 µm.

Selenoprotein S alleviates ROS‐induced oxidative damage and inflammation in SCI‐associated secondary damage [54, 55]. Therefore, we investigated the antioxidant effects of **Se@NADH** at 7 days post operation in vivo. The ROS bioluminescence of SCI mice models showed increased luminescence intensity of L‐012 (a marker of ROS) in lesion sites. In contrast, low ROS bioluminescence was observed in the **Se@NADH** treatment group, indicating that **Se@NADH** effectively eliminated ROS from lesion sites. Moreover, selenoprotein S expression were also upregulated in the **Se@NADH** treatment group, compared to the injury and other treatment groups (Figure 7A–C). Then, we investigated the anti‐inflammatory properties of **Se@NADH** in the spinal cord of mice. First, IL‐6 and IL‐1β levels in damaged regions of the spinal cord were examined by specialized Elisa kits. In Figure 7D,E, IL‐6 and IL‐1β levels in the spinal cord were significantly suppressed after treatment with **Se@NADH**, compared with the injury group. Furthermore, the generation of inducible nitric oxide synthase (iNOS) and ionized calcium‐binding adapter molecule 1 (Iba‐1) were evaluated via immunofluorescence to assess the anti‐inflammatory abilities of **Se@NADH**. After treatment with **Se@NADH**, iNOS and Iba‐1 levels in lesion sites of mice significantly reduced, indicating that **Se@NADH** suppressed inflammation in the injured spinal cord tissues (Figure 7F–H). The ability of **Se@NADH** to reduce apoptosis following SCI was confirmed by TUNEL (terminal deoxynucleotidyl transferase‐mediated deoxyuridine triphosphate nick end labeling)–DAPI double staining assay. In Figure 7I,J, the injury group exhibited representative apoptotic features, however, **Se@NADH** reduced neuronal apoptosis in the damaged region. Moreover, the result of H&E shows that cytoplasmic loss around the glomeruli in renal tissue and several physalides in liver tissue were observed in the injury group, while no obvious changes occurred in other organs (Figure S14). These results show that **Se@NADH** suppressed the injury and apoptosis of neurons.

## 3 Conclusions

There is an urgent need to investigate reagents with exceptional physicochemical properties for the treatment of SCI, to meet the current clinical application objectives. Therefore, we successfully designed and synthesized a functional **Se@NADH** nanodrug system which can confer neuroprotection and neuroregenerative capabilities by improving mitochondrial function and eliminating ROS to reduce oxidative damage and apoptosis of neurons. This smart design alleviated the instability of NADH under physiological conditions and achieved ROS‐responsive drug release. **Se@NADH** also inhibited inflammation in injured spinal cord tissues by restraining the activation of astrocytes and expression of proinflammatory cytokines with high safety. **Se@NADH** treatment decreased the formation of glial scars, accelerated neuronal regeneration, recovery of the electrophysiological traces and improved the survival of axons and myelin sheaths, which effectively improved the motion function of mice with SCI. Detailed analysis of the BMS score and CatWalk system, as additional functional behavioral tests, revealed significant improvements in movement, balance, and physical coordination in mice with SCI who received **Se@NADH** treatment. Together, this study not only provides an ultrasensitive redox‐responsive nanosystem of **Se@NADH** which can improve the mitochondrial function but also sheds light on the neuroprotective application mechanisms against SCI.

## Conflicts of Interest

The authors declare no conflicts of interest.

## Supporting information



Supporting Information

## Data Availability

The data that supports the findings of this study are available in the supplementary material of this article.
