# Supplementary material for: Dual‐Responsive Multi‐Functional Silica Nanoparticles With Repaired Mitochondrial Functions for Efficient Alleviation of Spinal Cord Injury
Source: Exploration (Beijing). 2025 Feb 6;5(3):270012. doi: 10.1002/EXP.70012 (PMC12199316; doi:10.1002/EXP.70012)
Supplement: Supplementary file 1 — Supporting Information [file EXP2-5-270012-s001.docx]

**Supplementary information**

Dual-responsive multi-functional silica nanoparticles with repaired mitochondrial functions for efficient alleviation of spinal cord injury

Guibin Gao^1#^, Juanjuan Li^1#^,Yanming Ma^3#^, Min Xie^2^, Jianxian Luo^2^, Ke Wang^2^, Cheng Peng^2^, Hua Yang^2^, Tianjun Chen^2^, Guowei Zhang^2^, Jiang Ouyang^1*^, Hongsheng Lin^2*^, Zhisheng Ji^2*^

1. Department of Dermatology, the Second Affiliated Hospital of Guangzhou Medical University; Guangdong Key Laboratory of Urology and Department of Urology, the First Affiliated Hospital of Guangzhou Medical University Guangzhou; Guangzhou Institute of Cancer Research, the affiliated Cancer Hospital & School of Biomedical Engineering, Guangzhou Medical University, Guangdong, 510120, China.

2. Department of Orthopedics, The First Affiliated Hospital, Jinan University, Guangzhou, Guangdong, 510632, China.

3. Honghui Hospital, Xi'an Jiaotong University

E-mail: ouyangjiang1989@gmail.com (J. Ouyang), tlinhsh@jnu.edu.cn (H-S Lin), tzhishengji@jnu.edu.cn (Z-S Ji)

**EXPERIMENTAL SECTION/METHODS**

**Synthesis**

**Synthesis of Se@NADH**

BTESePD and Se NP were synthesized as previously described. ^1^

To load NADH, a Se NP stock solution was created by sonicating 50 mg of Se NP in 3 mL of deionized water until fully suspended. Next, 8 mg of NADH was added to 1 mL Se NP stock solution, and the resulting mixture was shaken at 500 rpm at 4 °C for 12 h and the centrifuged. The precipitate was collected and suspended in deionized water. The amount of NADH adsorbed was calculated based on absorbance of the solution at 340 nm.

**Responsive release of nanoparticles**

The **Se@NADH** disintegration was evaluated by subjecting the samples to continuous vibration at 37°C in ultrapure water containing simulated intracellular concentrations of GSH (5 mM) and ROS (H_2_O_2_, 100 μM). After 2 days of incubation, the samples were examined using TEM.

**ABTS free radical scavenging assay**

We further performed the ABTS experiment to assess the oxidation resistance capacity of NADH and **Se@NADH**. Briefly, the ABTS free radical (ABTS^•+^) was generated through the reaction of manganese bioxide solution with the ABTS stock solution as previously reported.^2^ A series of concentration of NADH and **Se@NADH** were reacted with ABTS^•+^ free radical, and the absorbance at 734 nm was measurement within 60 min using a cell imaging multi-mode reader (Cytation 5, BioTek Instruments Inc.).

**Primary culture of hippocampal neurons and cell viability assay**

Primary hippocampal neurons were extracted as described previously. ^3^ Briefly, hippocampi were isolated from rat pups at postnatal 0 day and incubated in the DMEM including 10 % FBS and 10 % Ham's F-12 with glutamine (Gibco, Carlsbad, CA). They were then digested with trypsin and treated with bovine serum albumin (BSA) to terminate the digestion. The digested tissues were rinsed and rubbed gently. Thereafter, individual neurocytes were seeded at 20,000 cells/mL in 35-mm Petri dishes (Costar, Cambridge, MA). The nutrient solution was replaced with DMEM without elementary nutrients and containing 5 % B-27 supplement (Gibco). The cells were cultured for 24 h at 37^o^C, under 95 % O_2_ and 5% CO_2_. After the cells adhered to the plate, they were treated with different drug concentrations for another 24 h. Cytotoxicity toward cells was tested using the CCK8 assay.

**Detection of the inflammatory factors in the spinal cord**

The content of the inflammatory factors, including IL-6 and IL-1β in the spinal cord was measured using ELISA kits following the manufacturer’s instructions. ATP production were tested in line with the manufacturer’s protocol.

**Mitochondrial membrane potential (MMP) measurement, ROS detection, and ROS evaluation**

The MMPs were tested using the JC-1 Assay Kit (Beyotime, China) and the fluorescence intension (red and green) was measured. The ROS Assay Kit (Beyotime, China) was employed to evaluate ROS levels based on fluorescence intensity of DCFH-DA and DHE following the manufacturer’s protocol.

**Morphological analysis of mitochondria**

The morphology of mitochondria in HT-22 cells was examined using co-cultivation materials for the Mitotracker Red (100 nM) for 30 min following the manufacturer’s instructions. Mitochondrial morphology was examined and analyzed using confocal microscope and Image J (with Fiji MiNA plugin) similar to a previous study. ^4^

**Immunofluorescence assay**

The spinal cord tissues were treated with 4 % polyoxymethylene to settle them, and then made transparent with 0.3 % Triton X-100 before being sealed with 5 % BSA. Next, secondary antibodies and DAPI dye were applied to the spinal cord tissues after incubating them with specific primary antibodies overnight at 4 ^◦^C. Finally, the results were visualized using a fluorescence microscope.

**Western blot assay**

Proteins of spinal cord tissues were homogenized and treated with RIPA lysis (Beyotime, China). The concentration of proteins was determined using BCA kit (Beyotime, China). The proteins samples were separated using SDS-PAGE gels, and transferred to PVDF membranes. The membrane was blocked with a blocking buffer (Beyotime, China) for 1 h at room temperature and incubated with the specified primary antibodies at 4 ^◦^C overnight. It was then washed with TBST buffer and incubated for 2 h with specified secondary antibodies. The proteins bands were analyzed using the PowerOpti-ECL detection system (ThermoFisher Scientific, USA).

**Surgical procedures**

The SCI model was established as previously reported.^5^ The mice were narcotized with pentobarbital sodium and spinal cord sections were obtained through T10 laminectomy. A 10 g rod was dropped on the revealed spinal cord from a height of 12.5 mm to create an injury. The muscle and skin were sutured using 6-0 nylon sutures. Penicillin was administered intramuscularly every day for one week to prevent infection. In addition, a handmade urinary bladder was used two times one day to release urine until urinary bladder function was restored.

**Basso mouse scale (BMS) behavioral analysis**

All female C57BR/CdJN mice (6 weeks) in this study were purchased from GemPharmatech Co., Ltd. and all the animal studies were conducted with the Institutional Animal Use and Care Committee of Jinan University approval (No. 20231110-06). Mice are housed in a specific-pathogen-free (SPF) laboratory environment, with a standard practice of keeping five mice per cage. The nanometer materials were injected through the tail vein, mice are administered medication starting on the second day after the completion of the modeling process, and various tests are conducted after a continuous three-day treatment (the complexes (10 mg/ kg) and NADH (4 mg/kg). The recovery of hindlimb function was detected by the BMS which tested paw placement, body location and balance, coordinate ability of front and rear limbs, hind-limb joint activities, and miss mouse's tip. The mice were placed in an open field and their behavior were recorded. The scores were recorded before surgery and at 1, 3, 7, 14, 21, 28, 35, 42, 49, 56, and 63 days after SCI.

**Footprint analysis**

The footprint, locomotor behavior as well as body balance were evaluated using the CatWalk XT system that was applied to assess functional renewal after SCI. The CatWalk XT system coupled with a confined track with a glass panel containing green LEDs was used to record using a high-speed digital camera placed below. Each animal treated with different drugs was passed through the corridor and three successive runs were recorded using the camera.^6^

**Electrophysiology testing**

Electrophysiological experiments were conducted on mice to assess motor-evoked potentials (MEPs) at 9 weeks post-injury. After anesthetization, a stimulation electrode, the grounding line, a documenting electrode, as well as the reference electrode were inserted into rostral ends of revealed spinal cord, subcutaneously, into the biceps femoris flexor cruris at a depth of 1.5 mm, distal tendon of the hind limb muscle, respectively. A single square wave (0.5 mA, 0.5 ms, 1 Hz) was used to induce MEPs, and peak-to-peak amplitudes were recorded to estimate hind limb nerve transmission function.

**Oxidative stress of the spinal cord**

Sodium salt chemiluminescent probe (L-012) (75 mg/ kg) was intravenously injected to evaluate the intensity of oxidative stress, and the results were displayed by the Bidu imaging system (Ms Lumina I. Perkinelmei. USAnce).

**Statistical analysis**

All statistical analyses were performed using GraphPad Prism 8.0 (GraphPad Software Inc., USA). Multivariate analysis and comparison between the two groups were conducted using the one-way or two-way ANOVA test and Student’s t-test, respectively. Data were presented as the mean ± standard deviation (SD) and differences at **P* < 0.05, ***P* < 0.01, and ****P* < 0.001 were considered statistically significant.


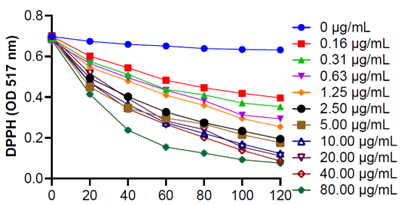


Figure S1. In vitro antioxidant activities of Se@NADH nanomaterials（0-80 μg/ml）as determined by DPPH assays.


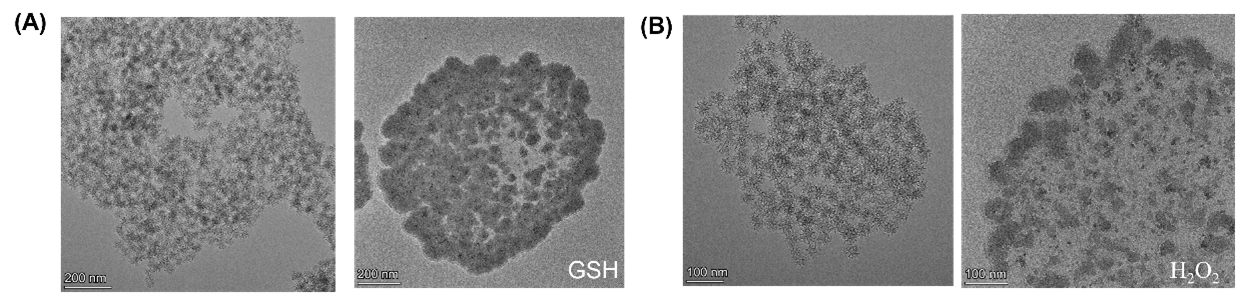


Figure S2.TEM images of **Se@NADH** after 2 d of incubation in the presence of 50 mΜ GSH (A) or 100 mM H_2_O_2_ (B).


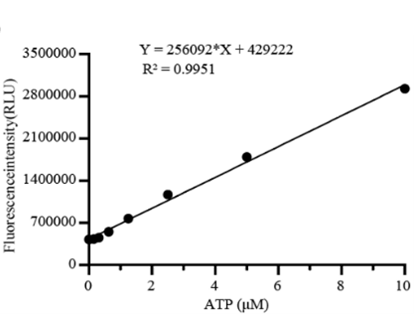


Figure S3. Standard curve for determine the production of ATP in cells.


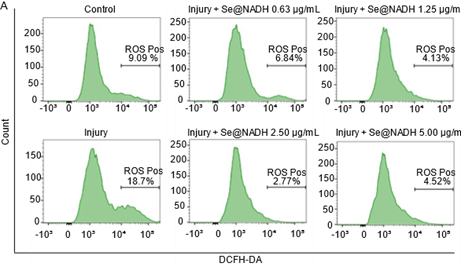


Figure S4. Detection of ROS levels treatment with Se@NADH by DCFH-DA in HT-22 cells by flow cytometry in indicated groups.


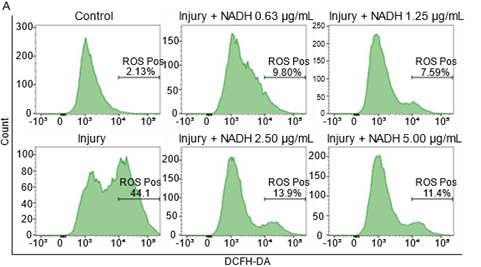


Figure S5 Detection of ROS levels treatment with NADH by DCFH-DA in HT-22 cells by flow cytometry in indicated groups.


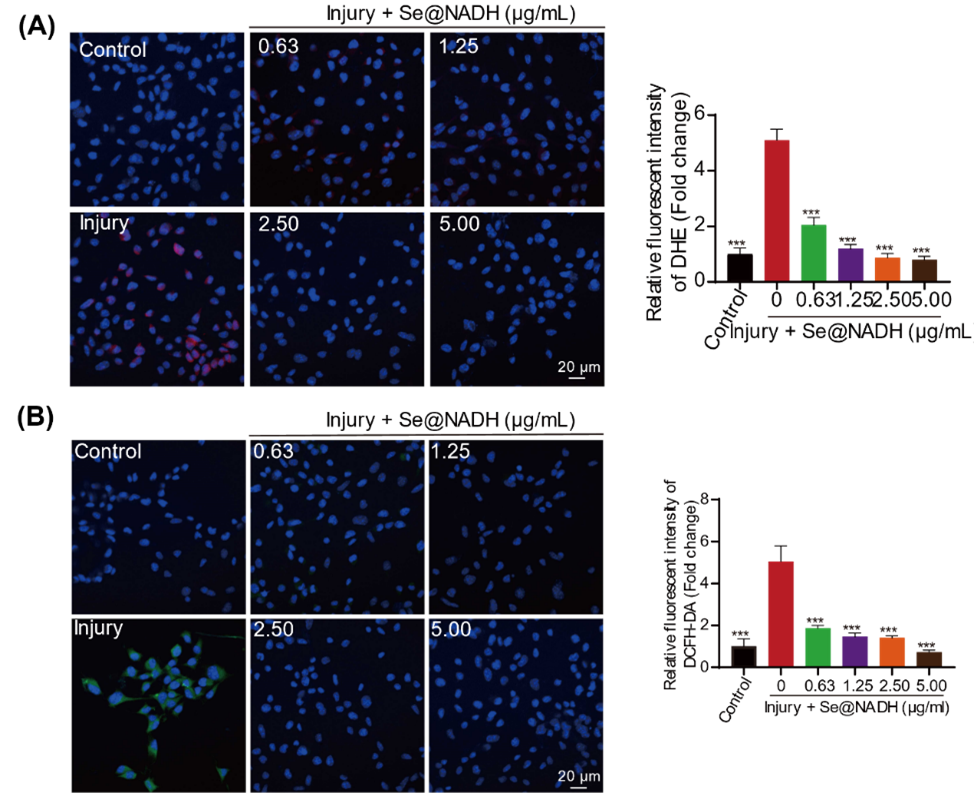


Figure S6. Detection and quantification of ROS levels treatment with Se@NADH by DCFH-DA in HT-22 cells by fluorescence staining with DHE or DCFH-DA in indicated groups.


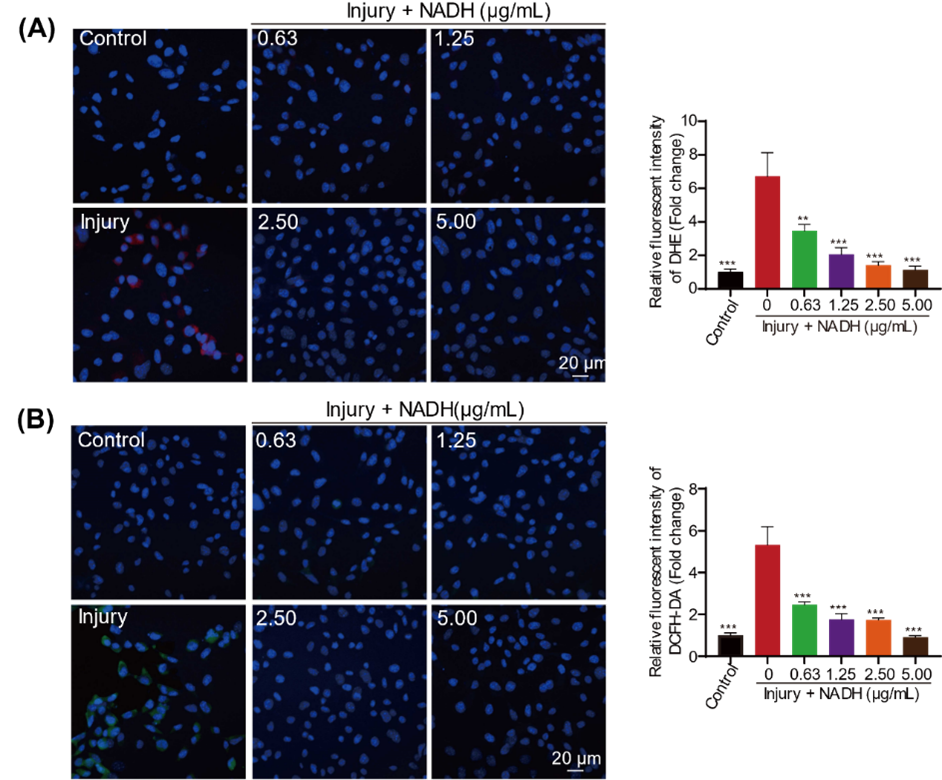


Figure S7. Detection and quantification of ROS levels treatment with NADH by DCFH-DA in HT-22 cells by fluorescence staining with DHE or DCFH-DA in indicated groups.


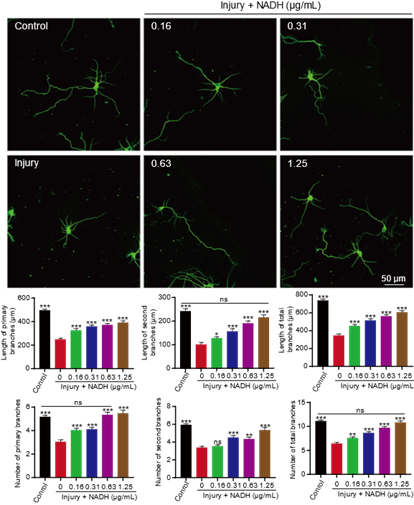


Figure S8. NADH promotes neurite outgrowth. (A) Representative images after co-cultured with NADH for 24 h in Hippocampal neuron. Scale bar: 50 μm. (B) above: The total length of primary and secondary branches, revealing that NADH promotes neurite outgrowth (n = 15/group). Below: The total number of the primary and secondary branches, revealing that NADH promotes branch formation (n = 15/group). Data are means ± SD, based on triplicate experiments. **P <* 0.05, ***P <* 0.01, and ****P <* 0.001.


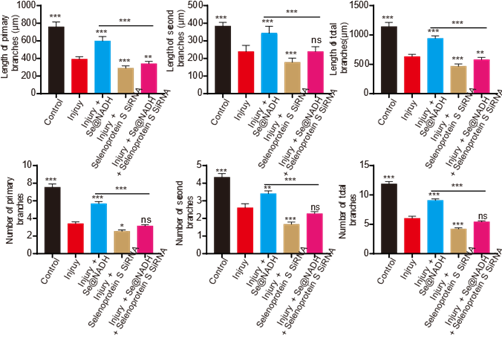


Figure S9. Above: The total length of primary and secondary branches, revealing that Se@NADH promotes neurite outgrowth (n = 15/group). Below: The total number of the primary and secondary branches, revealing that Se@NADH promotes branch formation (n = 15/group). Data are means ± SD, based on triplicate experiments. **P <* 0.05, ***P <* 0.01, and ****P <* 0.001.


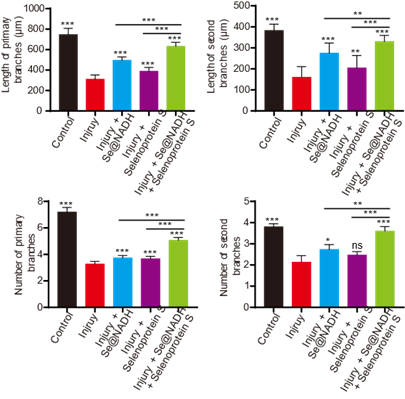


Figure S10. above: The total length of primary and secondary branches, revealing that Se@NADH promotes neurite outgrowth in overexpression selenoprotein S of Hippocampal neuron (n = 15/group). Below: The total number of the primary and secondary branches, revealing that Se@NADH promotes branch formation (n = 15/group). Data are means ± SD, based on triplicate experiments. **P <* 0.05, ***P <* 0.01, and ****P <* 0.001.


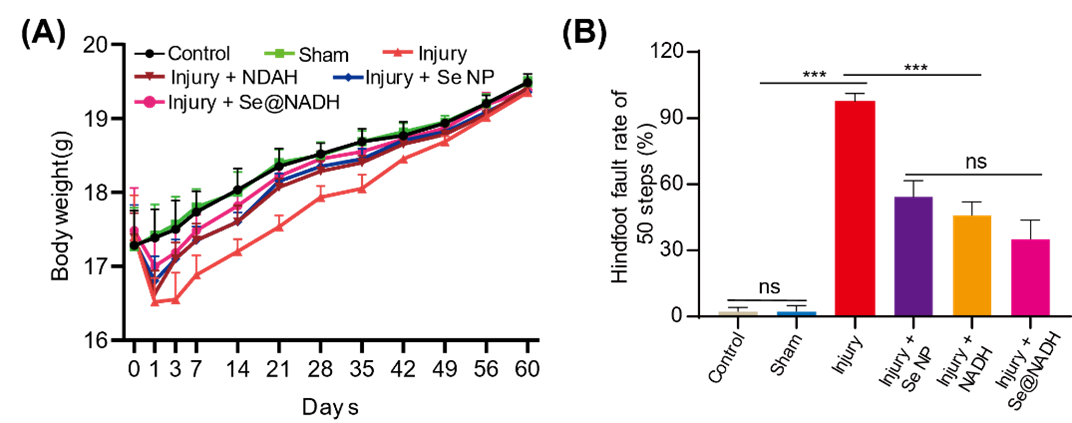


Figure S11 (A) The changed weight of mice after different treated. (B) The air stepping test was used to functionally measurement mice following 9 weeks post-injury.


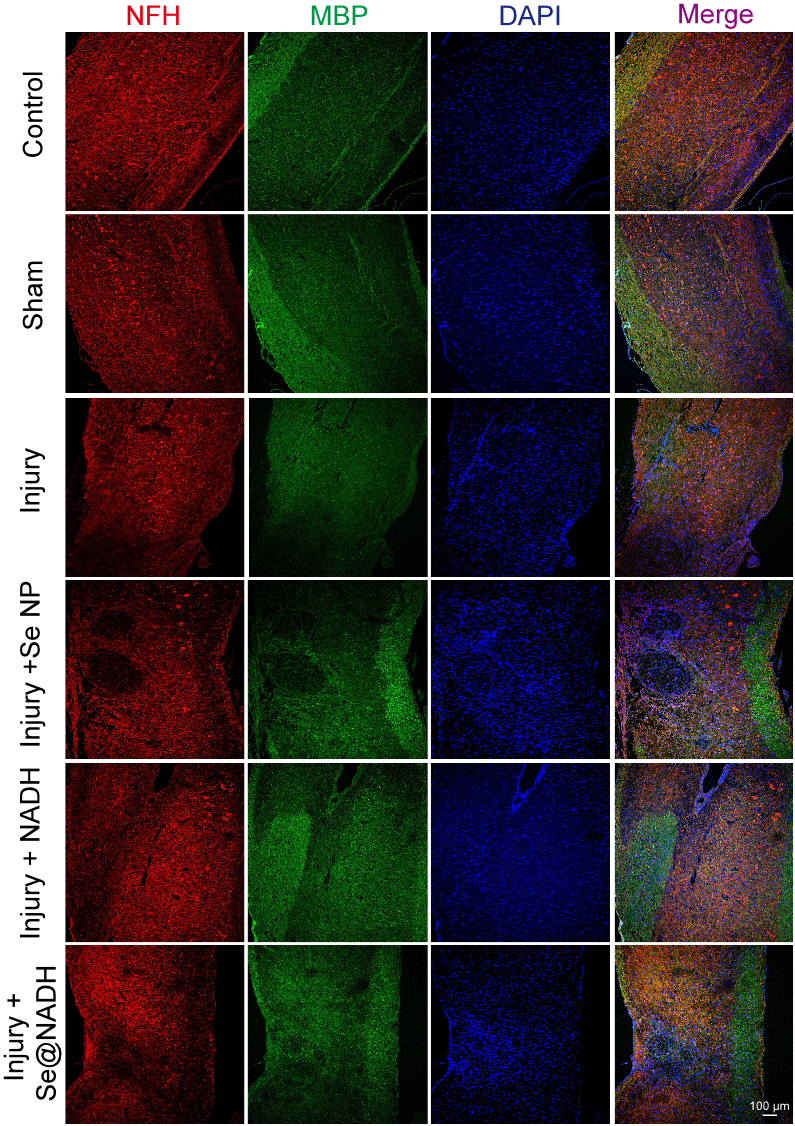


Figure S12. Immunofluorescence images of spinal cord tissue stained with anti-MBP (green) and anti-NFH antibodies(red), scale bar= 100 μm.


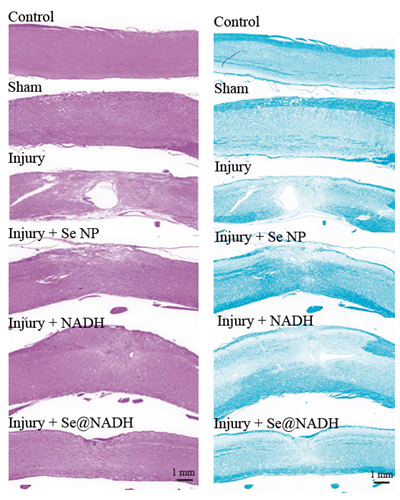


Figure S13. The images on the right are the spinal cord tissue after staining with H&E, the images on the right are the LFB staining of myelin sheaths.


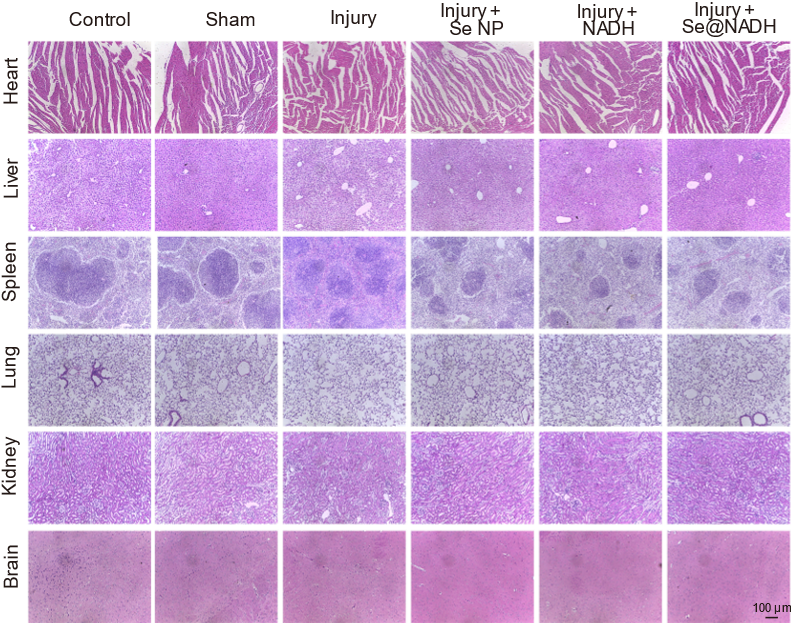


Figure S14. H&E staining of the heart, liver, spleen, lung, and kidney from different treatment groups in the SCI mice model.

[1] D. Shao, M. Li, Z. Wang, X. Zheng, Y.-H. Lao, Z. Chang, F. Zhang, M. Lu, J. Yue, H. Hu, H. Yan, L. Chen, W.-f. Dong, K.W. Leong, Bioinspired Diselenide-Bridged Mesoporous Silica Nanoparticles for Dual-Responsive Protein Delivery, Adv. Mater. 30(29) (2018) 1801198.

[2] Z.-S. Ji, G.-B. Gao, Y.-M. Ma, J.-X. Luo, G.-W. Zhang, H. Yang, N. Li, Q.-Y. He, H.-S. Lin, Highly bioactive iridium metal-complex alleviates spinal cord injury via ROS scavenging and inflammation reduction, Biomaterials 284 (2022) 121481.

[3] M. Tang, M. Wang, T. Xing, J. Zeng, H. Wang, D.-Y. Ruan, Mechanisms of unmodified CdSe quantum dot-induced elevation of cytoplasmic calcium levels in primary cultures of rat hippocampal neurons, Biomaterials 29(33) (2008) 4383-4391.

[4] M. Khacho, A. Clark, Devon S. Svoboda, J. Azzi, Jason G. MacLaurin, C. Meghaizel, H. Sesaki, Diane C. Lagace, M. Germain, M.-E. Harper, David S. Park, Ruth S. Slack, Mitochondrial Dynamics Impacts Stem Cell Identity and Fate Decisions by Regulating a Nuclear Transcriptional Program, Cell Stem Cell 19(2) (2016) 232-247.

[5] X.-J. Wang, C.-H. Peng, S. Zhang, X.-L. Xu, G.-F. Shu, J. Qi, Y.-F. Zhu, D.-M. Xu, X.-Q. Kang, K.-J. Lu, Polysialic-acid-based micelles promote neural regeneration in spinal cord injury therapy, Nano Lett. 19(2) (2019) 829-838.

[6] M. Leibinger, C. Zeitler, P. Gobrecht, A. Andreadaki, G. Gisselmann, D. Fischer, Transneuronal delivery of hyper-interleukin-6 enables functional recovery after severe spinal cord injury in mice, Nat. Commun. 12(1) (2021) 391.
